# From Onset and Prodromal Stage to a Life-Long Course of Schizophrenia and Its Symptom Dimensions: How Sex, Age, and Other Risk Factors Influence Incidence and Course of Illness

**DOI:** 10.1155/2019/9804836

**Published:** 2019-04-16

**Authors:** Heinz Häfner

**Affiliations:** Schizophrenia Research Group, Central Institute of Mental Health, Medical Faculty Mannheim/Heidelberg University, J5, 68159 Mannheim, Germany

## Abstract

The core symptoms of psychosis—delusions, hallucinations, and thought disorders—are not unique to the disorder traditionally called schizophrenia. They occur at the early stages of various brain diseases, too. Psychosis seems to be a preformed pattern of response of the human brain. Most schizophrenia onsets are marked by a prodromal stage extending over several years and producing the maximum of social consequences. Schizophrenia incidence shows a steep increase culminating at age 15 to 25 years in males. In females it reaches a first peak at age 15 to 30 years and a second, flatter peak at menopausal age (44-49 years). Thereafter, incidence declines to a plateau at later ages. Unlike what the findings of most large-scale epidemiological studies applying an upper age limit of 45 to 55 years suggest, schizophrenia is a disorder of all ages. The lifetime risk seems to be the same for both sexes. The lower incidence in premenopausal women is accounted for by the downregulating effect of oestrogen on dopamine receptors. This hormonal protective effect is antagonised by the genetic effect of a high familial load. In the long-term illness course, right-censored to 11.2 years following first admission, the number of psychotic relapse episodes ranges from 0 to 29 with a mean of 3. The positive symptom dimension produces the highest number of relapses and the shortest duration of exacerbations with a mean length of two months. The depressive and negative symptom dimensions show exacerbations extending over nearly six months on average. Following the first illness episode symptom scores decline sharply, reaching a plateau five years after first admission. Negative symptoms come to a plateau after 2 to 3 years in females and after 5 years in males. Depression is the most frequent type of symptom in the long-term course. In the light of these results urgent treatment issues will be discussed.

## 1. Introduction

Schizophrenia, defined as a disorder, is characterised by symptoms that distort or in part block some basic functions of the human mind, such as outer and inner perception and memory. In that respect schizophrenia is a disorder that hampers or distorts reality control during episodes of acute symptomatology and in a few cases also permanently.

Mental disorders involving loss of reality control can have serious consequences for both the affected individual and the community. For this reason, elucidating their causes has long been one of the main issues in psychiatric research. Since Emil Kraepelin's [1] first attempt to define schizophrenia as a unitary disorder by applying to it the diagnosis of “dementia praecox” we have seen over a hundred years of schizophrenia research. Given all these efforts we should actually know by now the underlying causes of the disorder and how to successfully treat it and the consequences it produces. But the truth is that as far as the symptoms leading to pronounced social disability and loss of quality of life, i.e., negative symptoms and cognitive impairment, are concerned, only modest progress has been achieved.

Defining the diagnosis Emil Kraepelin proceeded both from the main age of onset (praecox) and from the outcome (dementia) of the disease construct he called dementia praecox. The diagnostic features of a young age of onset, up to 45 years, and an unfavourable outcome frequently resembling dementia were eagerly accepted in those days and continued to be used in clinical practice. This changed partially after Eugen Bleuler's [2] concept of schizophrenia came to replace Kraepelin's dementia praecox, which happened more rapidly and widely in Europe than in the USA.

However, both of these criteria are wrong: a majority of patients suffering from so-called dementia praecox never develop genuine dementia even after long histories of illness, and illness onsets are fairly common beyond 60 years of age, too. These were the reasons why Eugen Bleuler [2], a Swiss psychiatrist, expanded the diagnosis and replaced “dementia praecox” by “schizophrenia”. But neither the term “schizophrenia” nor “split mind” provides an apt description of the true nature of the disorder. Split personality or dissociative personality disorder today denotes a condition in which the affected person adopts two or more distinct identities that each is unaware of the other. Unlike schizophrenia, a split personality does not involve psychosis.

Giving the disorder a new name Eugen Bleuler also introduced ancillary concepts intended to explain the pathology involved, such as autism, ambivalence, associative disturbances, none of which are specific for schizophrenia and hence have not qualified as proper descriptions. The same is true of the disease concepts and their underlying causes proposed after Bleuler. All this just reflects the fact that we simply do not know yet what schizophrenia really is.

## 2. Materials, Methods, and Results

As we set out to throw some light on this murky chapter of schizophrenia research, we first looked for findings and issues that, when replicated and resolved, were promising of providing insights into the fundamental nature of the disorder.

### 2.1. Sex Difference in Age of Onset

A fairly fundamental finding seemed to us to be what Kraepelin [3] and some other researchers after him had observed, namely that women tended to be a few years older than men at first admission for a diagnosis of schizophrenia [4]. There was no plausible explanation at hand for that phenomenon at that time yet. Looking for one, we first had to check whether this age difference at first admission was actually caused by a difference in age of onset. First-admission age depends not only on age at onset, but also on an array of environmental factors, such as age at first receiving the diagnosis, sociocultural and socioeconomic factors, on differences in health-care systems and on whether inpatient treatment is paid by patients themselves, a health insurance or the state.

Our first social hypothesis ran that incipient schizophrenia and its prodromal stage are perceived later in nonemployed women in their home environment than in employed men. At the time of our investigation, conducted in Denmark and the Mannheim area in Germany, women of childbearing age in paid work were clearly a minority in both places. We compared first-admission age between employed and nonemployed men and women on data from the national Danish and the Mannheim psychiatric case registers in cooperation with P. Munk-Jørgensen's team [5]. We found that at both study sites both groups of women, employed and nonemployed, were several years older than their male counterparts at first admission. This was a significant finding.

#### 2.1.1. Findings from IRAOS Data

**Figure 1:**
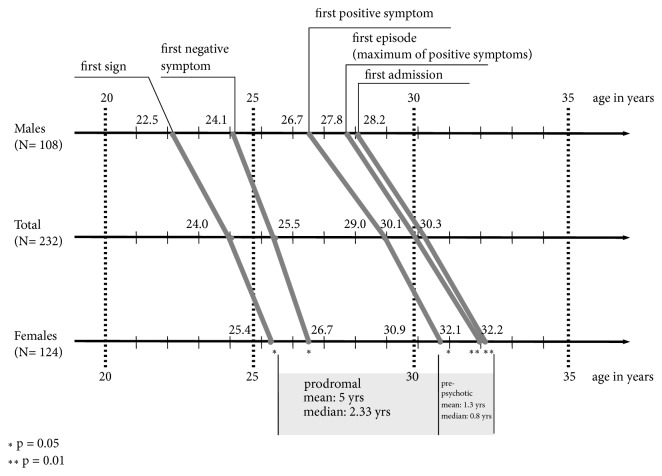
Mean age by 5 definitions of onset until first admission; ABC first-episode sample of broadly defined schizophrenia (N=232). Source: [8], modified.

Given this result, we then had to find a way of accurately determining age of schizophrenia onset. For this purpose we developed the “Interview for the Retrospective Assessment of the Onset and Course of Schizophrenia and Other Psychoses”, IRAOS [6, 7]. The instrument permits one to assess both the time of first-ever symptom onset and that of the actual illness onset. Producing retrospective data on the manifestation of signs of illness it helps to determine the age at which a person first meets the diagnostic criteria for schizophrenia as set out in the international classification systems. Using that instrument, we found a mean sex difference of 2.9 years in age at the appearance of the first-ever sign of illness and a difference of 3.6 years in age at the onset of the first psychotic symptom of any type. As Figure 1 shows, mean age at first admission was 28.2 years for males and 32.2 years for females (a difference of 4.0 years). Mean age at the climax of the first illness episode, defined by the maximum number of positive symptoms presented by the patients, was 27.8 years for males and 32.1 years for females (a difference of 4.3 years) [8]. All the definitions of illness onset yielded a sex difference fairly similar in size.

Astonishing about this result was that the sex difference did not emerge after the manifestation of the first psychotic episode, for example, as a corollary of the unfolding biological disease process, but was already there to a considerable extent when the very first signs of disorder appeared. This meant that looking for factors explaining the sex difference we had to focus on causes active at earlier stages and probably biological in nature.

#### 2.1.2. Transnational Comparisons

**Figure 2:**
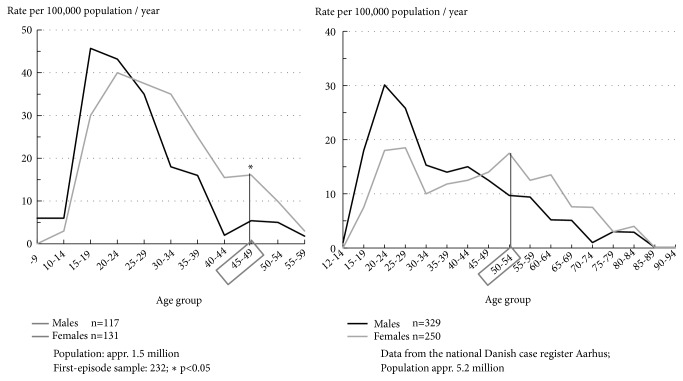
*Left:* ABC Schizophrenia Study: age at onset of schizophrenia (ICD-9: 295, 297, 298.3, 298.4) (first psychotic symptom) by sex;* right:* population-based first-admission rates for schizophrenia (ICD-8: 295) in Denmark 1976 by age and sex. Source: [9].

Since the IRAOS is a culture-neutral instrument, we were able to compare data on the sex difference in age of onset between different geographical areas, e.g., countries, political systems, and ethnicities. We started by analysing first-admission data on schizophrenia from the Danish national case register (1976) and data on first-episode cases from the German A (age) B (beginning) C (course) Schizophrenia Study (1987-1989). The two data sets were compiled using comparable criteria. The analysis was conducted in cooperation with our Danish colleagues [5, 9]. The results are presented in Figure 2.

The age distributions for males and females depicted on the left differ from those on the right in two respects: first, in the ABC Schizophrenia Study data on schizophrenia onset were collected for probands in age range 12 to 59 years from a population of about 1.5 million living in the area of the cities of Mannheim and Heidelberg, Rhine-Neckar District and eastern Palatinate, whereas the Danish case-register data stemmed from the whole of the Danish population of about 5.2 million without any age limit. The second difference was that, as we had expected, the age distributions for both sites showed a high first peak at early adult age—higher for males than females—and a second peak for females in menopausal age, but that second peak of onsets turned up some 5 years later, on average, in the Danish (Figure 2,* right*) than the German data (Figure 2,* left*). A difference of one year and a few months would have been reasonable, because the German data were based on age at the onset of the first psychotic symptom and the Danish data on age at first admission, but that did not explain the bulk (2-3 years) of the difference we found.

To explain the unexpected rest of that age difference in the pattern of schizophrenia onsets between the Danish and German females, we drew a randomized, stratified sample of 116 first-admission patients with a diagnosis of schizophrenia or related disorder from the Danish case register and obtained their case records from the hospitals where they had been treated. We did this in cooperation with our Danish colleagues and with the permission of the regional authorities [10, 11]. Using a case-note version of the IRAOS we determined for this sample 1) age at the onset of the first psychotic symptom and 2) age at fulfilling the diagnostic criteria for a schizophrenia diagnosis (i.e., the earliest point in time when the patients actually presented a sufficient number of symptoms qualifying them for the diagnosis according to the International Classification of Diseases, ICD-9). In this way we made sure that the Danish and Mannheim data were based on identical definitions of age at onset.

We did not expect to achieve the result we did: while 51% of the Danish males clearly meeting the diagnostic criteria for schizophrenia had received that diagnosis at discharge from hospital, the corresponding figure for females was only 32%. No such sex difference in age either at the appearance of the first psychotic symptom or at fulfilling the diagnostic criteria for schizophrenia emerged in the Mannheim data [10]. Hence, it was reasonable to assume that, compared with male patients, Danish psychiatrists—at that time they were mostly men—were less inclined to give their newly admitted female patients the unpopular diagnosis of schizophrenia. As the case-record analysis showed, they preferred milder, fuzzier diagnoses for their female patients, such as pubertal crisis, psychogenic psychosis, acute paranoid reaction or borderline. The bottom line of this hypothesis testing thus was that when identical diagnostic criteria (International Classification of Diseases, ICD-9) were applied to both the Danish and the Mannheim data the previously observed transnational age difference in the second, flatter peak in female schizophrenia incidence vanished and that peak for Danish females, too, fell in the same age group as in Mannheim, namely 45 to 49 years (s. Figure 2*, left*).

We then proceeded to test the transnational and transcultural stability of the sex difference in age of onset on a wider range of countries, because until then we had focused on two culturally fairly uniform European countries. A global comparison would have been unfeasible for lack of detailed enough data from a sufficiently large number of international epidemiological schizophrenia studies.

**Figure 3:**
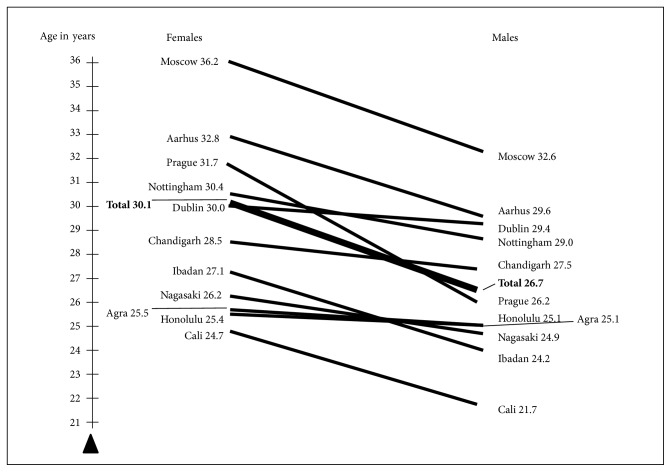
WHO Determinants of Outcome Study: mean ages at onset in the centres and the total sample (N=1,292). Source: [12].

For this reason we chose the then best possible access to international data for addressing this question [12]: in cooperation with N. Sartorius and A. Jablensky we analysed data from the WHO 10-country study (DOSMed) [13, 14]. The data had been collected using identical definitions and instruments at four sites in three low-income countries (Agra/India, Chandigarh/India, Cali/Columbia and Ibadan/Nigeria) and at seven sites in high-income countries (Aarhus/Denmark, Moscow/USSR, Honolulu/USA, Prague/CSFR, Nagasaki/Japan, Nottingham/England, and Dublin/Ireland). The mean first-contact age we found was 3.4 years higher for women than men, thus confirming our previous findings (Figure 3) [12]. And at most of the study sites age of onset was clearly higher for women, as Figure 3 demonstrates. The rare exceptions (e.g., in Honolulu/USA and Agra/India the sex difference in first-contact age was a mere 0.3 and 0.4 years respectively) were mostly accounted for by flawed data collection methods.

Venturing a conclusion from these results, it is reasonable to expect that a sex difference consisting in women's being a few years older than men at schizophrenia onset is bound to emerge anywhere in the world provided that sound study methods are being applied.

A British meta-analysis of 46 selected epidemiological studies yielded a significant, but only small sex difference of about a year in age of onset of schizophrenia [15]. The sex difference was considerably larger in epidemiological studies using the DSM (Diagnostic and Statistical Manual of Mental Disorders) criteria than in those based on the ICD criteria. In the latter case the sex difference even failed to attain significance. A reverse sex difference similar in size was reported for Asian countries. A careful look at the meta-analysis, however, shows that this result was based only on a very small number of publications from a handful of countries and that the local populations studied were small. A study from Ethiopia and one study from India even reported a higher age of onset for males. Two of the Indian studies included in the analysis in particular showed severe flaws in the way the probands' age and the first-admission samples' representativeness were determined. In the first [16] of these two Indian studies about half of the patients' dates of birth were not officially documented and had to be based on the information provided by the patients and/or their family members. For reasons of local culture, especially unmarried women, if their age was not documented, tended to be reported to be younger than 18 years old [B.N. Gangadhar, personal communication]. Probably the second Indian study [17], too, was marred by these problems of determining the probands' age, but the authors make no mention of it. This second study was based on the case records of two rather small samples (N=70 each) admitted to the same mental-health centre from two different states. While patients from the one state showed a sex difference, i.e., a higher age of onset for males than females, those from the other state showed no significant difference. Shortcomings in the representativeness of the samples studied also apply to some of the other studies included in the meta-analysis without being made allowance for. Hence, the conclusion to draw is that the results of this meta-analysis cannot be regarded as truly valid.

A few other reviews focusing on the sex difference in age of onset of schizophrenia in various smaller national populations [4, 18–21] have also reported a lower age at schizophrenia onset for men than women and thus, despite the problem of small numbers, almost invariably confirmed our finding.

### 2.2. Distribution of Onsets across the Life-Cycle

The strategy we had thus far adopted in our research activities, i.e., consistent hypothesis testing and formulating new questions and new hypotheses on the basis of the results achieved, had turned out successful. Sticking with this strategy we started a long-term research project, the afore mentioned ABC Schizophrenia Study [22, 23], which we hoped would take us a few steps closer to attaining our goal of elucidating some aspects of the disease construct called schizophrenia. In this project, applying the approach described we investigated the course of schizophrenia unfolding from onset to prepsychotic prodromal stage to long-term course and also some of the intrinsic and extrinsic risk and protective factors involved.

#### 2.2.1. Male-Female Comparisons 

**Figure 4:**
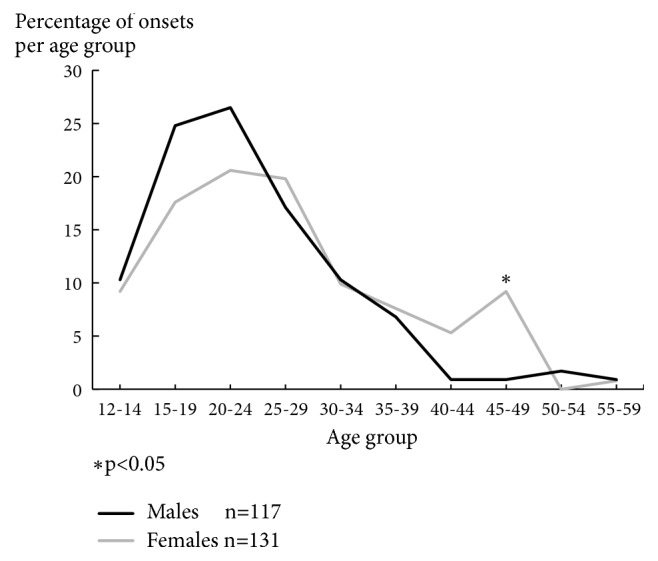
Distribution of age at onset of schizophrenia (first-ever sign of mental disorder) by sex; ICD 9-295, 297, 298.3, 298.4 (ABC Schizophrenia Study). Source: [25].

So far we have discussed age at illness onset in males and females on the basis of various consecutive definitions of that event (manifestation of the first sign of illness, of any type, manifestation of the first negative symptom, of the first positive symptom, of the maximum of positive symptoms and first admission) over the course of incipient disorder. Since schizophrenia is a disorder of all ages, we should take a look at the sex-specific age distributions at illness onset across the life-cycle. In the ABC Schizophrenia Study we analysed the age distribution at illness onset for males and females up to age 60 years and obtained an intriguing overall picture [24]:

As Figure 4 shows, in accordance with the epidemiological findings from Germany and Denmark depicted in Figure 2, male onsets start increasing steeply at around age 15 years and reach a maximum at early adult age in age range 15 to 24 years. That maximum is followed by a steady decline, which ends in a plateau extending from age 40 to 60 years.

In contrast, female onsets are somewhat slower to increase in youth. They reach a first, slightly flatter peak compared to males at age 15 to 30 years, as already demonstrated in Figure 2. The ensuing continuous decline is followed by a second peak, somewhat flatter than the first one, at age 45 to 49 years, again, as we had previously seen on the Danish data. In this age group female onsets differ significantly from male onsets in frequency. It is not until after age 50 to 54 years that the frequency of female onsets finally shows a plateau.

Since our ABC study sample was limited to age 60 years and younger, we studied another sample of 1109 consecutive first admissions different from the ABC sample and not limited by age. Included in that sample were first admissions for a diagnosis of schizophrenia spectrum disorder (ICD-9) to the Central Institute of Mental Health, a mental-health care facility located in Mannheim, Germany, and serving the city and its surrounding region [22]. We found that onsets of schizophrenic psychosis do occur in both men and women in old and very old age, too.

A look at the incidence of schizophrenia spectrum disorders over the entire life-cycle shows that the lifetime risk seems to be approximately the same for both men and women. In contrast, most of the large international epidemiological studies report a higher lifetime risk for males than females (*see below*). The reason is that all these studies have used 45 to 55 years as an upper age limit, thus partly or completely excluding the higher incidence rates of postmenopausal women. The most pronounced male-female differences in incidence fall in certain phases of life: a higher male incidence in the first half of life and a higher female incidence in menopausal age in the second half of life. The second peak of female onsets means that women finally catch up on incidence in the second half of life. As a result, both sexes end up with nearly the same lifetime risk of currently 13.21/100,000 for men and 13.14/100,000 for women at age 60 years [25]. As stated, these figures are in discrepancy with quite a number of epidemiological studies on the topic in that those studies report a higher lifetime risk for men [26–31].

#### 2.2.2. Incidence and Symptoms in Younger versus Older Age Groups

**Figure 5:**
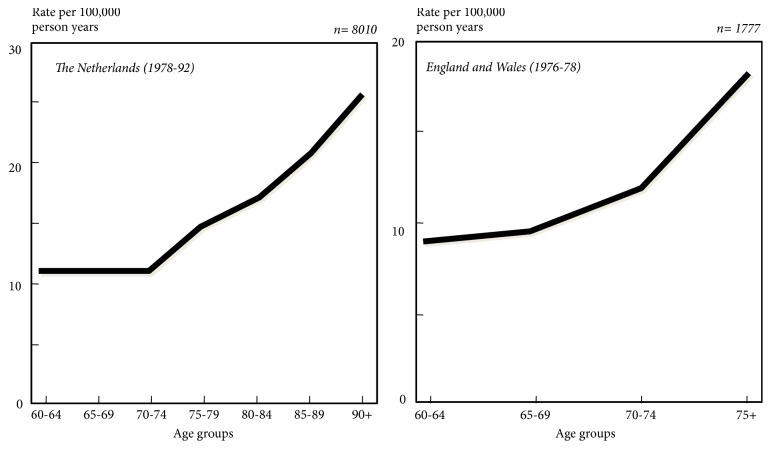
Annual first-contact rates for nonaffective psychoses in the elderly population (>60 years); based on case-register data. Source: [32], modified.

If it is true that schizophrenia can manifest at any age, from youth to old age, we should make sure that in all these cases we are really dealing with the same disorder. Certain descriptive differences have emerged in selected population-based epidemiological studies not excluding higher ages of onset, e.g., in that conducted by van Os and colleagues [32]. That study focused on schizophrenia onsets in the Netherlands in the years 1978 to 1992 and in England and Wales from 1976 to 1978. This methodologically sophisticated population-based study demonstrated on the two data sets that even in age ranges 60 to 75 years and older and 60 to 90 years and older there is a considerable quantitative risk of onset of schizophrenia spectrum disorder (Figure 5). A symptom-related analysis showed that paranoid delusions and secondary delusions were more frequent in first-onset cases at advanced age than in younger age groups.

This crude indication that first-onset cases might differ in their symptoms depending on age prompted us to compare symptom presentation in first episodes of schizophrenia spectrum disorder over the entire life-cycle. We based our analysis on five-year age of onset groups extending from 15-19 to 75 years and older for males and females in our second study sample of 1109 first admissions for schizophrenia [22].

**Figure 6:**
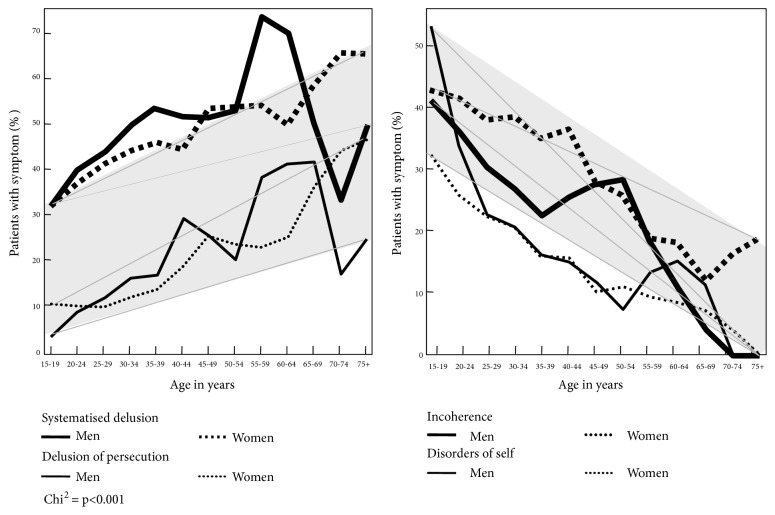
Psychotic symptoms showing significant age trends; based on data from CIMH (N=1,109)* (CIMH: Central Institute of Mental Health)*. Source: [33].

The bulk of the symptoms we looked at in the first illness episodes did not display any substantial age differences, apart from childhood-onset and early-youth-onset schizophrenia. Nor did the occurrence of the first-rank symptoms described by Kurt Schneider and included in the ICD-9 schizophrenia diagnosis (specific delusions, hallucinations and thought disorders) depend on age. However, there were two symptom dimensions that ran counter to this pattern: systematised delusions and delusions of persecution, i.e., paranoid and delusional phenomena (Figure 6). The frequency of persecutory delusions rose from near zero at young age to almost 30% for males and to over 40% for females in old age. A similar pattern was observed with systematised delusions: they increased in frequency from initially some 30% to almost 50% for men and to over 60% for females respectively. The frequencies of incoherence and disorders of self fell from high values in the youngest group to almost zero in the oldest. These opposite age-related trends were linear in type and significant.

The symptoms that showed markedly different frequencies in old age—systematised delusions in particular, but also paranoid delusions—are complex in nature and contain a cognitive component of processing primary symptoms such as severe anxiety. They are complex, because they have multiple functional components. Usually, they also have a cognitive component, which means that through these symptoms persons experiencing them try to cope for example with severe anxiety arising from a delusional belief of being harmed by external causes (paranoid), or the symptoms are embedded in a comprehensive delusional system constructed as an explanation for fearful events or experiences. A further characteristic of such cognitive coping by way of systematised delusions is externalisation, i.e., attributing unpleasant experiences, personal failures and mishaps or feelings of guilt to external causes. This type of behaviour is quite common in elderly persons not diagnosed with schizophrenia, too. Obviously, age seems to have a considerable impact on the core symptoms of schizophrenia.

In contrast, the clinical picture of childhood-onset schizophrenia is characterised by hardly any or no symptoms possessing a clear-cut cognitive component. Typical of the disorder at this age are primary or elementary symptoms, such as diffuse, at times very severe states of anxiety, fluctuating attention disorders, profoundly altered perception of self and others, elementary distortions of self-experience, such as believing that thoughts are being inserted from outside into one's mind, and symptoms of mental confusion [34, 35]. The sense of immediacy in combination with considerable mental disorganisation reflected in these symptoms is probably a corollary of the still rather instable structure of the self at an age when mental, especially cognitive, development is still in progress and brain maturation, which underlies mental maturity, has not yet come to an end either. This developmental-psychological phenomenon is the reason why childhood-onset and early-onset psychosis can exert such a disruptive effect on the basic functions of personality.

As a consequence of these factors, the onset of schizophrenia in childhood—very rare—and in youth seems to be associated with particularly severe socioeconomic consequences in the further course of the disorder [34–40].

At a higher age, when personality has matured and attained a more stable structure of the self and the cognitive functions have fully developed, the pathological process of schizophrenia can no longer upset the core structures and central functions of personality to any greater extent. As a consequence, there will be less mental disorganisation and social impairment and more symptoms of cognitive and projective coping, i.e., by attributing unpleasant or fearful experiences to outer causes. In addition, due to decreasing emotional intensity in old age, late-onset schizophrenia tends to produce symptoms characterised less by emotional dynamism.

### 2.3. Explaining the Sex Difference in Age of Onset

Let us return to the sex difference in age of onset. Since it seems reasonable to assume that women are a few years older than men at any stage of incipient schizophrenia, except at a higher age, we are faced with the question of what causes this fundamental difference in disease development.

The graphs illustrating the age distributions of men and women at schizophrenia onset in Figures 2 and 4 suggest that maturational factors govern the dynamics of the pathological process in a different way in men and women up until middle age. After that age, including menopausal age in women, the system steering the manifestation of the disorder produces a lifetime risk very similar in size for both sexes. This age distribution, especially the peak in female incidence at menopausal age, points to a causal hypothesis.

Underlying women's distribution of schizophrenia onsets could be an oestrogen effect. The secretion of this sex hormone increases steeply in puberty and declines rapidly as soon as women reach menopause. The effect of the male sex hormone testosterone on the organism, too, increases sharply at young age, but it shows no rapid decline comparable to that of oestrogen at menopausal age.

This could mean that the flatter increase in female incidence at young age and the second peak at menopausal age point to a kind of protective effect capable of warding off illness onset in some women for a certain period of time. As soon as these women reach menopause, the protective effect comes to an end and the illnesses warded off until then manifest. A conceivable alternative explanation for men's lower age of onset and more severe symptoms in the first half of life might be that testosterone is capable of speeding up schizophrenia manifestation, in other words: it exerts an effect opposite to that of oestrogen. We tested both hypotheses in animal experiments.

#### 2.3.1. Protective Effect of Oestrogen

We tested how the two sex hormones influence dopaminergic behaviour. The effect of four-week oestrogen applications was compared with testosterone applications in three groups of animals (rats): (1) Group 1 received dopaminergic stimulation through apomorphine, (2) Group 2 comprised sham-stimulated, and (3) Group 3 nonstimulated, but placebo-treated animals. It was exclusively in the oestrogen-treated group that a neuroleptic-like blockade of dopaminergic behaviours could be observed. A histochemical analysis showed that oestrogen down-regulates D_2_-receptors [41, 42], which is a sign of oestrogen exerting an antipsychotic effect. In contrast, testosterone showed no such effect.

The conclusion to draw from these results is that oestrogen essentially accounts for the sex difference in age of schizophrenia onset. The oestrogen hypothesis has since been confirmed experimentally in a direct way, too. Treatment trials have demonstrated that medium to high doses of estradiol as an adjunct to classic, treatment-guideline-conform antipsychotic therapy has an additional antipsychotic effect in women of childbearing age diagnosed with schizophrenia or schizoaffective disorder [43–47].

#### 2.3.2. Impact of Familial Load on Sex Difference in Age of Onset

**Figure 7:**
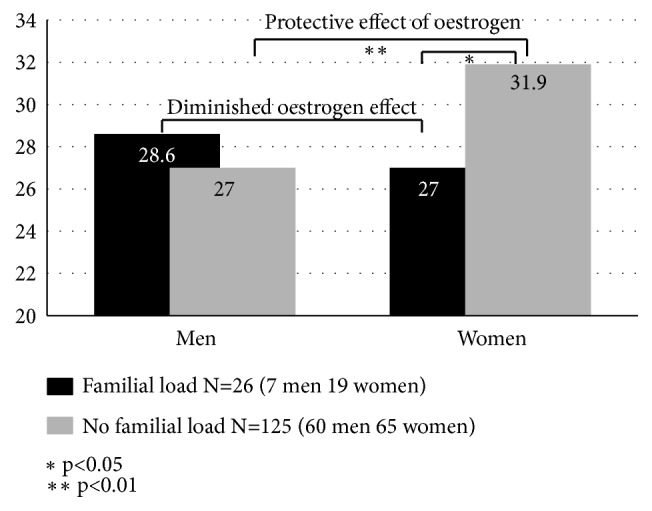
Age at first psychotic symptom by gender and familial load; ABC first-episode sample of schizophrenia (N=232). Source: [51].

Conclusive as this result may seem there is still the question of whether the oestrogen effect is the only conceivable, neurophysiologically effective mechanism capable of bringing about the sex difference in age of schizophrenia onset. There are good reasons for asking this question, for, according to the studies published by DeLisi et al. [48] and Albus and Maier [49], cotwins and siblings of persons diagnosed with schizophrenia show no or a smaller sex difference in age of onset. We tested the hypothesis that genetic load is capable of antagonising the protective effect of oestrogen. We compared adolescent first-degree relatives of persons suffering from schizophrenia with adolescents free from such familial load. And indeed, probands with familial schizophrenia showed no significant sex difference in their age of onset, and this result was accounted for solely by a considerably decreased age of onset in female—not male—probands with familial schizophrenia [50] (Figure 7).

At 4.9 years the difference in age of onset between women with familial load and women free of such load was slightly greater than the age difference between the corresponding male groups, on whose age of onset familial load exerted no significant effect. We concluded from this result that the schizophrenia-boosting impact of genetic load must be more powerful than the schizophrenia-reducing hormonal effect.

### 2.4. Age-Related Risk Factors

Having explained the sex difference in age of schizophrenia onset until menopausal age we were intrigued to find out how age and other major risk factors might influence age at onset in men and women in the second half of life. We examined our ABC sample of first illness episodes and found that with increasing age the impact of both familial load for schizophrenia spectrum disorder and that of pre-, peri-, and postnatal brain damage declines steadily in both men and women [50]. The diminishing impact of these risk factors, however, does not seem to contribute to explaining the opposite age trends in symptomatology, i.e., the increase in paranoid symptoms and the decline in disorders of the self and diffuse delusional states with growing age. The fact that the effect of genetic load and environmental risk factors on illness onset is at its greatest at a young age probably also contributes to explaining why, besides increased vulnerability attributable to yet incomplete maturation, a schizophrenia onset in childhood or youth is characterised by more severe, diffuse pathology, as previously discussed.

Rational cognitive coping with the disorder by means of systematised and paranoid delusions and a tendency to externalisation, as already stated, probably represent types of reaction typical of old and very old age. They are triggered by secondary risk factors such as hearing loss and a subjective feeling of loneliness [52, 53]).

An important age-related factor is the socially negative behaviour of young males. Compared with men aged 40 years and older and women of any age younger men diagnosed with schizophrenia show a significantly higher frequency of aggressive behaviour, self-neglect and some other types of socially adverse behaviour [54]. This same age-related pattern of socially negative behaviour also emerged in our control sample of men not suffering from schizophrenia we drew from the population of our study area. This means that men with schizophrenia present socially negative behaviour in excess of what is regarded as a “normal” personality trait in their healthy peers. However, in both groups of men, those with and those without schizophrenia, socially negative types of behaviour become considerably less frequent after age 40 years. From that age on the further social course of schizophrenia in men is influenced by the disease process rather than a variety of age-related behaviours.

### 2.5. Course of Schizophrenia

While focusing on age- and sex-related aspects of illness onset and symptomatology we have thus far considered aspects of illness course only fleetingly. Let us now explore how the prodromal, short-, medium-, and long-term course of schizophrenia spectrum disorder depend on inherent (disease-related) and external (environmental) factors. We will also take a look at the social course, governed by patients' illness-related social impairment on the one hand and by the type and amount of social consequences, i.e., changes in socioeconomic, occupational and familial conditions, patients suffer over the course of their illness, on the other hand.

#### 2.5.1. Social Course

**Figure 8:**
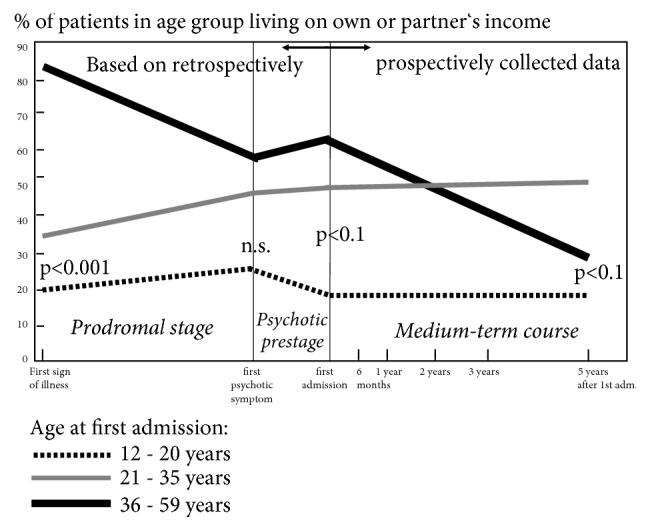
Social course of schizophrenia as based on economic independence (smoothened curves) in the early and medium-term illness course in three groups of patients aged 12-20, 21-35, and 36-59 years at inclusion in study (= first admission), ABC follow-up sample (n=115). Source: [60], modified.

In the ABC study we analysed how age and social status at illness onset influence patients' further social course. In terms of the frequency patients experience social decline during illness course, early-onset patients, who develop the disorder before finishing school and/or occupational training, suffer only modest social decline in the further illness course. Instead, since an early illness onset impedes social development and becomes reflected in lack of attainment of common steps of socioeconomic ascent, they rather suffer social stagnation. In contrast, individuals who have already run through the usual stages of social attainment before falling ill at middle or a higher age are at risk of suffering pronounced social decline. Young adults who have finished school and occupational training and have a promising occupational career occupy an intermediate position in terms of social decline [9, 55] (see Figure 8).

In old age, when people have reached retirement and ceased to work and the changes in socioeconomic conditions typical of this age have already taken place, the risk of experiencing social decline as a consequence of schizophrenia onset is fairly small. The situation looks different and shows a lot of variation as far as the other concomitant social characteristics of illness course, such as number and quality of social contacts and range and availability of leisure activities, are concerned. A precondition for not suffering socioeconomic loss due to illness in old age is that there are pension schemes and/or a health-care system in place that guarantee an adequate living, treatment and care—as in Western societies—through state provision and/or private insurance. Under such conditions there is hardly any risk of suffering objective social decline in old age. The situation is different in countries without adequate provision for old age: there the proportions of poor social courses of schizophrenia tend to be the largest among people falling ill in old age after working life has ended [56–59].

In Figure 8 we have depicted how patients' economic independence, which usually reflects a financially stable employment relationship, develops in age range up to 59 years, i.e., the usual working age, as an indicator of social course and outcome in schizophrenia.

As mentioned, a maximum of onsets of schizophrenia and schizophrenia-like disorders occurs between 15 and 25 years of age for men and between 15 und 30 years of age for women. In the Western culture these are the periods of life characterised by the most pronounced individual socioeconomic development. As a consequence, the earlier illness onset in males tends to be associated with more pronounced social consequences, whereas the later onset in females leads to less severe social consequences. This sex difference is reflected in patients' level of social competence and even influences medium-term (5-year) social course and outcome in male patients. Another factor with an unfavourable impact on young men's social competence in the early and medium-term illness course is the fact mentioned above that schizophrenia clearly compounds normal socially negative male behaviour.

**Figure 9:**
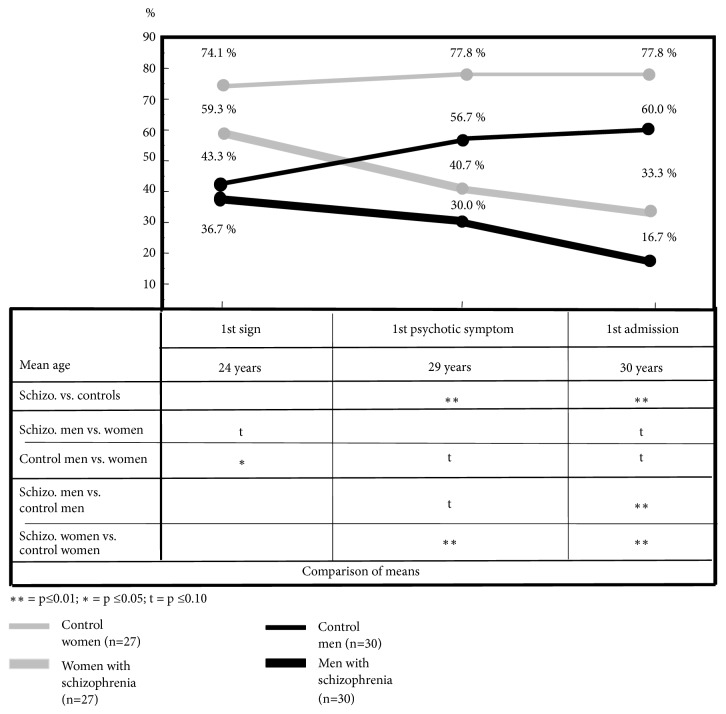
Marriage or stable partnership. Source: [61].

To give an example for a social course from the prodromal stage of schizophrenia extending from onset to first admission we have depicted in Figure 9 the proportions of patients and controls either married or living in a stable partnership (> 1 year).

As the figure shows, larger proportions of healthy women than women with schizophrenia or healthy men marry or enter a stable partnership at early adult age. Part of this finding is attributable to the fact that at the time of our study, on average, women in Germany were some years younger than their male counterparts when getting married. With increasing age, the proportion of healthy men married or living in a stable partnership, too, increases markedly, but clearly falls short of the percentage of healthy women. In contrast, the corresponding proportions of both men and women with schizophrenia show a steady decline resulting in significant differences to their healthy peers at first admission [54].

In the further course of illness, not depicted in the Figure, divorce rates for both male and female patients are on the rise. But larger proportions of female than male patients are capable of finding new partners and remarrying, while growing proportions of their male counterparts are living alone. Underlying this sex difference are normal sex-related behaviours, and this also applies to young male patients' socially negative behavioural traits. Women, on average, have better communication skills and more sex appeal than men have. But schizophrenia permanently reduces their bonding abilities, too, with a result of high divorce rates among female patients.

#### 2.5.2. Long-Term Social Course

As far as the long-term social course is concerned, we reexamined probands from our ABC first-episode sample an average of 12.3 years after first admission where ever we could reach them, at home, hospital, or residential home. The results on the social course of schizophrenia we obtained were encouraging. Just under a third of the probands (32.7%) had a regular job, as was the case at first admission [23]. However, this result was not genuinely representative, because due to the sample's upper age limit of 59 years at first admission some of the probands had already retired prior to the follow-up. 24 of the original sample of 232 patients fully assessed had died (8 of them clearly and further 7 probably of suicide) [23].

#### 2.5.3. Long-Term Symptom-Related Course

For a detailed analysis of long-term illness course we right-censored the probands' histories of illness at 11.2 years (= 134 months), the shortest span between first admission and follow-up (range: 11.2-14.6 years). In this way we obtained identical durations of illness for all probands [23].

The frequency of psychotic relapse episodes (a psychotic relapse was defined as a two-week period of deteriorating psychotic symptoms preceded and followed respectively by four weeks in which symptoms were either absent or reduced) showed a mean of three per patient over the total course of 11.2 years. With a minimum of 0 and a maximum of 29 relapses there was a lot of variation indicating a high degree of heterogeneity in the illness courses [23] and no perceivable association with adherence to antipsychotic treatment.

Besides the course of schizophrenia as such we also analysed how the individual symptom dimensions—positive, negative, depressive, and manic—evolved. Based on means, symptoms declined continuously on these dimensions; however, the numbers for the manic dimension were too small to be included in this analysis. The decline started at first admission and extended over up to five years at the most and differed in speed. Thereafter, all the symptom dimensions showed a plateau-like course without any further decline or increase in symptoms. No significant sex differences could be found either on the positive or the depressive symptom dimension. On the negative symptom dimension women reached a plateau sooner than males did, i.e., in two to three years after first admission compared to four to five years for men. From that point on not only did all the symptom dimensions, but also the course of schizophrenia as such show no essential variation in the sense of either deterioration or further recovery [23]. This important finding indicates that after a five-year history of illness schizophrenia and its core symptom dimensions tend to have grown fairly stable without undergoing further improvement or deterioration in the future course.

Besides tracing the course of the overall symptom dimensions mentioned and counting the individual illness episodes on the basis of a defined duration we also took a detailed look at the exacerbations irrespective of their duration. At a median of two months positive symptoms showed the shortest exacerbations and negative and depressive symptoms the longest at a median duration of almost five months respectively [23].

To sum up, characteristic of the long-term symptom-related course of schizophrenia is an initially high degree of heterogeneity and, after several years of illness, considerably less heterogeneity both in the overall clinical presentation and on the core symptom dimensions. All the pathological dimensions have in common a course that first shows a sharp decline in the core symptoms, which is followed, after a five-year history of illness, by more or less stable mean symptom frequencies. The only exception to this pattern is displayed by positive symptoms in that they decline more rapidly after illness onset and settle on a plateau as soon as two to three years after that event. A further exception is that on the negative symptom dimension female patients start displaying a plateau one to two years earlier than male patients do. The only other sex differences observable in the long-term course of schizophrenia are limited to age at illness onset up until menopausal age and the consequences that sex difference produces in the medium-term illness course and the social course until old age.

### 2.6. Genetic Aspects, Age of Onset, and Symptom Severity

Since an early age of onset is to a great extent genetically determined while a very late onset of schizophrenia spectrum disorder shows no clear-cut genetic component, the question arises again whether age of onset and severity of first-episode schizophrenia might generally be associated with a more or less pronounced familial load.

Asarnow [62] reviewed 24 high-risk studies. Most of these studies examined children who had a biological parent diagnosed with schizophrenia. Three of the samples included were characterised by behavioural abnormalities indicative of the probands' being at risk for schizophrenia. The 24 studies differed in their quality. Irrespective of their methodological shortcomings the studies showed that children and adolescents with a high familial-genetic risk tended to show signs of “neurointegrative” problems, social impairment and early symptoms, especially in middle childhood and adolescence, and moderate deficits in attention, information processing, neuromotor function and social behaviour. This means that a high genetic load is associated with an excess risk for an early illness onset, as we showed on the ABC Schizophrenia sample, and with considerably more severe psychopathology compared with late-onset schizophrenia, underlying which is a smaller genetic load.

The enormous advances achieved in molecular-genetic research in the last few years have considerably improved our understanding of the genetic transmission of schizophrenia risk, despite the failure to settle the question conclusively. A long time ago it had become clear that only a tiny fraction of schizophrenias are transmitted by the Mendelian laws of inheritance. In such cases the risk for schizophrenia is usually associated with further neurobiological deficits such as mental retardation, peripheral malformations etc. These findings, however, do not provide pointers to the modes of transmission in the overwhelming majority of genetically determined cases of schizophrenia spectrum disorder.

The complex mode of transmission of schizophrenia risk is governed by susceptibility or risk genes, each of which accounts only for a tiny fraction, less than 2%, of the total morbid risk [63, 64]. These susceptibility genes are widespread in the general population, too, where they occur in different constellations and vary in frequency. The overwhelming majority of these risk genes existing in the genome of healthy individuals, however, lack the power to cause the manifestation of schizophrenia.

Systematic genome-wide analysis have recently yielded quantitative information on the number of risk genes and thus made it possible to validate the mathematical hereditary estimates proposed on the basis of epidemiological twin and family studies. These analyses have revealed a considerably lower heredity, but we do not yet know how to explain this difference.

Gene editing, a newly developed method of isolating and removing single genes by means of restriction enzymes, has enabled researchers to conduct selective, targeted operations on DNA chains. Using this method it has been possible to remove pathogenic amino acid sequences resulting from mutations. First the pathogenic genes are cut out using the technique of gene surgery and then a piece of the organism's own normal DNA is inserted in the gap as a replacement for the pathogenic sequence.

This type of gene surgery is currently being practised on plants and isolated animal species. In humans its application will be limited to mononuclear hereditary diseases, and in rare cases it has already been applied successfully. It is hardly conceivable that a sufficient number of the vast array of susceptibility genes for schizophrenia thus far identified could be modified by means of gene surgery in order to prevent the genetic risk for schizophrenia from materialising. Gene surgery is simply not practical in schizophrenia because it is impossible to remove and replace such a large number of risk genes—each of which carries only a weak effect—and not to produce undesirable side-effects.

## 3. Conclusions

The main factor underlying schizophrenia risk seems to be genetic in nature. But schizophrenia is not a hereditary disorder. The mode of transmission involved is a complex one. The manifestation of schizophrenia is governed by a combination of intrinsic and extrinsic risk factors, which not only produce psychosis as the core syndrome, but also determine the actual disease process, including negative symptoms and social impairment.

The social consequences patients suffer as a consequence of their negative symptoms and social impairment vary with the cultural environment, while the core symptoms largely show no cultural variation.

The core syndrome of schizophrenia—psychosis—is not a discrete disorder with a unitary cause. It can be caused by multiple factors. For example, a psychosis can be developed as a reaction to certain intoxications (e.g., cannabis, cocaine, and mescaline) or be caused by acute severe brain dysfunction (confusional state, delirium). It can also occur as a preliminary symptom of neurobiological degenerative processes, for example, at early stages of Alzheimer's disease, or be caused by central lesions in multiple sclerosis and in rare hereditary diseases such as Huntington's chorea.

Schizophrenia is a traditional notion with a more than a century-long history. Today, as a result of our accumulated knowledge, it is better seen in a more differentiated way and broken down to its components. The core disorder, constituted by delusions and hallucinations, is a type of dysfunctional unit, probably representing a preformed pattern of reaction of the human brain. As mentioned, it can be triggered by a variety of brain disorders or cerebral processes. In these cases the only way to treat it is to target the underlying pathology. By medications capable of downregulating the overactive functional network of transmitters and synapses merely a symptomatic treatment of the core syndrome can be achieved, but hardly any alleviation of the negative and cognitive symptoms and socioeconomic consequences.

### 3.1. The Nature and Course of Schizophrenia

The core syndrome of the disease construct called schizophrenia—hallucinations, delusions, and thought disorders—occurs in the same form and its lifetime risk shows approximately the same frequency anywhere in the world where these questions have been studied. Underlying the symptom pattern of psychosis is probably one of the rare preformed reaction patterns of the human brain. This seems to be true of the reaction patterns of depression and dementia, too. The underlying causes of psychosis—different types of neurobiological dysfunctioning in the brain—are heterogeneous. Their causal treatment is rarely possible and can only be achieved by treating the underlying pathology provided that it can be identified. The only treatment available for psychosis in schizophrenia is symptomatic therapy. The core pathology in schizophrenia is associated with a variety of aetiological risk factors that lead to the manifestation of the core syndrome and some of its components, such as depression or social impairment.

These aetiological risk factors comprise in the first place primary risk factors such as genetic load, pre-, peri-, and postnatal complications and early infections of the brain, e.g., childhood virus encephalitis. Further risk factors are age, sex, and a few socially relevant behavioural predispositions, e.g., young men's socially negative behaviour, a phenomenon encountered both in persons with schizophrenia and likewise in healthy individuals.

The multiplicity of the causes underlying schizophrenia is reflected in the great variety of types of illness course. In around a fifth of all cases schizophrenia produces just one psychotic episode and no socioeconomic consequences. A small proportion of schizophrenia cases are progressive in type and associated with impairment in several domains of life as well as slightly reduced brain volumes.

Most persons diagnosed with schizophrenia show mild brain volume reduction, which is locally accentuated (hippocampus, temporal, and prefrontal cortical regions) and already there at illness onset. In a small proportion of patients brain volume reduction progresses slowly, mostly accompanied by growing cognitive deficits, but no dementia comparable to Alzheimer's type.

As stated, the main risk factor for schizophrenia is genetic in type, but its impact grows the weaker the later illness onset occurs. Genes carrying a high risk for schizophrenia are rare and are usually attributable to chromosomal defects or other similar severe anomalies. A majority of patients with schizophrenia possess risk genes, but each of these genes accounts only for 2% of the total risk at most [63, 64], and they are also widespread in the general population. Genome-wide analyses have counted more than a thousand such risk genes.

There are also multiplicity of environmental factors that enhance the risk for schizophrenia.

The first psychotic episode is usually preceded by a prepsychotic prodromal stage unfolding over several years. It is followed by a shorter psychotic prodromal stage prior to the first treatment contact. Most of the unfavourable consequences the disorder leads to occur at these early stages of illness. A lengthy untreated prodromal period is a predictor of an unfavourable illness course.

The treatment of choice for psychosis caused by an underlying condition is a therapy targeted at that condition. There is no causal therapy for schizophrenia available yet. The current medical treatment of psychotic symptoms is capable of reducing acute psychotic symptoms and of preventing psychotic relapses. The greatest problem in the treatment of schizophrenia is the lack of effective therapy for negative symptoms and cognitive deficits, the main causes of the social consequences associated with the disorder. A further problem is posed by depressive mood, the most frequent symptom patients present over the illness course. It, too, requires targeted therapy.

## Data Availability

Previously reported data from the ABC Schizophrenia Study and related studies were used to support this study and are available in articles published by Heinz Häfner, as first or coauthor in various psychiatric journals. These prior studies and datasets are cited at relevant places within the text as [5–12, 18, 22–25, 33, 41, 42, 50, 51, 54, 55, 60, 61].

## References

[1] Kraepelin E. (1896). *Psychiatrie, 5. Aufl*.

[2] Bleuler E., Aschaffenburg G. (1911). Dementia praecox oder Gruppe der Schizophrenien. *Handbuch der Psychiatrie*.

[3] Kraepelin E. (1909-1915). *Psychiatrie, 8. Aufl*.

[4] Angermeyer M. C., Kühn L. (1988). Gender differences in age at onset of schizophrenia. *European Archives of Psychiatry and Clinical Neuroscience*.

[5] Häfner H., Riecher A., Maurer K., Löffler W., Munk-Jørgensen P., Strömgren E. (1989). How does gender influence age at first hospitalization for schizophrenia? A transnational case register study. *Psychological Medicine*.

[6] Häfner H., Riecher-Rössler A., Hambrecht M. (1992). IRAOS: an instrument for the assessment of onset and early course of schizophrenia. *Schizophrenia Research*.

[7] Häfner H., Löffler W., Maurer K., Riecher-Rössler A., Stein A. (2003). *IRAOS. Interview for The Retrospective Assessment of The Onset and Course of Schizophrenia and Other Psychoses*.

[8] Häfner H., Gattaz W. F. (1995). Geschlechtsunterschiede bei der schizophrenie. *Der Gynäkologe*.

[9] Häfner H., Hambrecht M., Löffler W., Munk-Jørgensen P., Riecher-Rössler A. (1998). Is schizophrenia a disorder of all ages? A comparison of first episodes and early course across the life-cycle. *Psychological Medicine*.

[10] Löffler W., Häfner H., Fätkenheuer B. (1994). Validation of Danish case-register diagnosis for schizophrenia. *Acta Psychiatrica Scandinavica*.

[11] Lützhøft J. H., Skadhede S., Fatkenheuer B. (1995). Symptom assessment in casenotes and the clinical diagnosis of schizophrenia. *Psychopathology*.

[12] Hambrecht M., Maurer K., Häfner H., Sartorius N. (1992). Transnational stability of gender differences in schizophrenia? An analysis based on the WHO study on determinants of outcome of severe mental disorders. *European Archives of Psychiatry and Clinical Neuroscience*.

[13] Sartorius N., Jablensky A., Korten A. (1986). Early manifestations and first-contact incidence of schizophrenia in different cultures. *Psychological Medicine*.

[14] Jablensky A., Sartorius N., Ernberg G. (1992). Schizophrenia: manifestations, incidence and course in different cultures. a world health organization ten-country study. *Psychological Medicine Monograph*.

[15] Eranti S. V., MacCabe J. H., Bundy H., Murray R. M. (2013). Gender difference in age at onset of schizophrenia: a meta-analysis. *Psychological Medicine*.

[16] Murthy G. V. S., Janakiramaiah N., Gangadhar B. N., Subbakrishna D. K. (1998). Sex difference in age at onset of schizophrenia: discrepant findings from India. *Acta Psychiatrica Scandinavica*.

[17] Gangadhar B. N., Panner Selvan C., Subbakrishna D. K., Janakiramaiah N. (2002). Age-at-onset and schizophrenia: reversed gender effect. *Acta Psychiatrica Scandinavica*.

[18] Hambrecht M., Maurer K., Häfner H. (1992). Gender differences in schizophrenia in three cultures. results from the collaborative WHO disability study. *Social Psychiatry and Psychiatric Epidemiology*.

[19] Messias E. L., Chen C., Eaton W. W. (2007). Epidemiology of schizophrenia: review of findings and myths. *Psychiatric Clinics of North America*.

[20] Ochoa S., Usall J., Cobo J., Labad X., Kulkarni J. (2012). Gender differences in schizophrenia and first-episode psychosis: a comprehensive literature review. *Schizophrenia Research and Treatment*.

[21] Morgan C., John S., Esan O. (2016). The incidence of psychoses in diverse settings, INTREPID (2): a feasibility study in India, Nigeria, and Trinidad. *Psychological Medicine*.

[22] Häfner H., Maurer K., Löffler W. (1998). The ABC schizophrenia study: A preliminary overview of the results. *Social Psychiatry and Psychiatric Epidemiology*.

[23] Häfner H., Maurer K., an der Heiden W. (2013). ABC Schizophrenia study: an overview of results since 1996. *Social Psychiatry and Psychiatric Epidemiology*.

[24] Häfner H., Riecher-Rössler A., an der Heiden W., Maurer K., Fätkenheuer B., Löffler W. (1993). Generating and testing a causal explanation of the gender difference in age at first onset of schizophrenia. *Psychological Medicine*.

[25] Häfner H., Maurer K., Löffler W., Riecher-Rössler A. (1991). Schizophrenie und Lebensalter. *Nervenarzt*.

[26] Lewine R. R. J., Nasrallah H. A. (1988). Gender and schizophrenia. *Handbook of Schizophrenia*.

[27] Iacono W. G., Beiser M. (1992). Are males more likely than females to develop schizophrenia?. *The American Journal of Psychiatry*.

[28] McGrath J., Saha S., Welham J., El Saadi O., MacCauley C., Chant D. (2004). A systematic review of the incidence of schizophrenia: the distribution of rates and the influence of sex, urbanicity, migrant status and methodology. *BMC Medicine*.

[29] Kirkbride J. B., Fearon P., Morgan C. (2006). Heterogeneity in incidence rates of schizophrenia and other psychotic syndromes: findings from the 3-center AeSOP study. *Archives of General Psychiatry*.

[30] Thorup A., Waltoft B. L., Pedersen C. B., Mortensen P. B., Nordentoft M. (2007). Young males have a higher risk of developing schizophrenia: a Danish register study. *Psychological Medicine*.

[31] van der Werf M., Hanssen M., Köhler S. (2014). Systematic review and collaborative recalculation of 133 693 incident cases of schizophrenia. *Psychological Medicine*.

[32] van Os J., Howard R., Takei N., Murray R. (1995). Increasing age is a risk factor for psychosis in the elderly. *Social Psychiatry and Psychiatric Epidemiology*.

[33] Häfner H., Ehrenreich H., Gattaz W. F., Louza M. R., Riecher-Rössler A., Kulkarni J. (2006). Oestrogen – A protective factor in schizophrenia?. *Current Psychiatry Reviews*.

[34] Remschmidt H. (2009). *Schizophrenia in Children and Adolescents*.

[35] Eggers C. (2011). *Schizophrenie des Kindes- und Jugendalters*.

[36] Eggers C., Bunk D. (1997). The long-term course of childhood-onset schizophrenia: A 42-year followup. *Schizophrenia Bulletin*.

[37] Asarnow J. R., Tompson M. C., McGrath E. P. (2004). Annotation: childhood-onset schizophrenia: clinical and treatment issues. *Journal of Child Psychology and Psychiatry and Allied Disciplines*.

[38] Gillberg C., Remschmidt H. (2009). Epidemiology of early onset schizophrenia. *Schizophrenia in Children and Adolescents*.

[39] Hollis C., Rapoport J., Weinberger D. R., Harrison P. J. (2011). Child and adolescent schizophrenia. *Schizophrenia*.

[40] Kendhari J., Shankar R., Young-Walker L. (2016). A review of childhood-onset schizophrenia. *Focus*.

[41] Häfner H., Behrens S., De Vry J., Gattaz W. F. (1991). Oestradiol enhances the vulnerability threshold for schizophrenia in women by an early effect on dopaminergic neurotransmission. *European Archives of Psychiatry and Clinical Neuroscience*.

[42] Gattaz W., Behrens S., De Vry J., Häfner H. (1992). Östradiol hemmt Dopamin-vermittelte Verhaltensweisen bei Ratten – ein Tiermodell zur Untersuchung der geschlechtsspezifischen Unterschiede bei der Schizophrenie. *Fortschritte der Neurologie · Psychiatrie*.

[43] Kulkarni J., de Castella A., Downey M., Häfner H. (2002). Clinical estrogen trials in schizophrenia. *Risk and Protective Factors in Schizophrenia – Towards a Conceptual Model of the Disease Process*.

[44] Kulkarni J. A., de Castella A., Fitzgerald P. B. (2008). Estrogen in severe mental illness: a potential new treatment approach. *Archives of General Psychiatry*.

[45] Kulkarni J., Gavrilidis E., Hayes E., Heaton V., Worsley R. (2014). Special biological issues in the management of women with schizophrenia. *Expert Review of Neurotherapeutics*.

[46] Kulkarni J., Gavrilidis E., Wang W. (2014). Estradiol for treatment-resistant schizophrenia: a large-scale randomized-controlled trial in women of child-bearing age. *Molecular Psychiatry*.

[47] Akhondzadeh S., Nejatisafa A. A., Amini H. (2003). Adjunctive estrogen treatment in women with chronic schizophrenia: a double-blind, randomized, and placebo-controlled trial. *Progress in Neuro-Psychopharmacology & Biological Psychiatry*.

[48] DeLisi L. E., Bass N., Boccio A., Shields G., Morganti C., Vita A. (1994). Age of onset in familial schizophrenia. *Archives of General Psychiatry*.

[49] Albus M., Maier W. (1995). Lack of gender differences in age at onset in familial schizophrenia. *Schizophrenia Research*.

[50] Könnecke R., Häfner H., Maurer K., Löffler W., an der Heiden W. (2000). Main risk factors for schizophrenia: increased familial loading and pre- and peri-natal complications antagonize the protective effect of oestrogen in women. *Schizophrenia Research*.

[51] Häfner H. (2003). Gender differences in schizophrenia. *Psychoneuroendocrinology*.

[52] Howard R., Jeste D., Weinberger D. R., Harrison P. J. (2011). Late-onset schizophrenia. *Schizophrenia*.

[53] Linszen M. M., Brouwer R. M., Heringa S. M., Sommer I. E. (2016). Increased risk of psychosis in patients with hearing impairment: Review and meta-analyses. *Neuroscience & Biobehavioral Reviews*.

[54] Häfner H., Maurer K., Miller T., Mednick S. A., McGlashan T., Libiger J., Johannessen J. O. (2001). The prodromal phase of psychosis. *Early Intervention in Psychotic Disorders*.

[55] Häfner H., Maurer K., Löffler W., an der Heiden W., Könnecke R., Hambrecht M., Häfner H. (2002). The early course of schizophrenia. *Risk and Protective Factors in Schizophrenia*.

[56] Ran M., Xiang M., Chan C. L. (2003). Effectiveness of psychoeducational intervention for rural Chinese families experiencing schizophrenia. *Social Psychiatry and Psychiatric Epidemiology*.

[57] Ran M., Chen S., Chen E. Y. (2011). Risk factors for poor work functioning of persons with schizophrenia in rural China. *Social Psychiatry and Psychiatric Epidemiology*.

[58] Ran M. S., Weng X., Chan C. L.-W. (2015). Different outcomes of never-treated and treated patients with schizophrenia: 14-year follow-up study in rural China. *The British Journal of Psychiatry*.

[59] Ran M., Chan C. L., Ng S., Guo L., Xiang M. (2015). The effectiveness of psychoeducational family intervention for patients with schizophrenia in a 14-year follow-up study in a Chinese rural area. *Psychological Medicine*.

[60] Häfner H., Maurer K., an der Heiden W. (2013). Schizophrenie – eine einheitliche Krankheit?. *Nervenarzt*.

[61] Häfner H., Häfner H., Wolpert M. (1996). The epidemiology of onset and early course of schizophrenia. *New Research in Psychiatry*.

[62] Asarnow J. R. (1988). Children at risk for schizophrenia: converging lines of evidence. *Schizophrenia Bulletin*.

[63] Kahn R. S., Sommer I. E., Murray R. M. (2015, article no 15067). Schizophrenia. *Nature Reviews Disease Primers*.

[64] Schizophrenia Working Group of the Psychiatric Genomics Consortium (S. Ripke et al.) (2014). Biological insights from 108 schizophrenia-associated genetic loci. *Nature*.

